# An intersectional analysis providing more precise information on inequities in self-rated health

**DOI:** 10.1186/s12939-020-01368-0

**Published:** 2021-02-03

**Authors:** Maria Wemrell, Nadja Karlsson, Raquel Perez Vicente, Juan Merlo

**Affiliations:** 1 Unit for Social Epidemiology, Department of Clinical Sciences Malmö, Lund University, Jan Waldenströms Street 35, 205 02 Malmö, Sweden; 2 Department of Gender Studies, Lund University, Lund, Sweden; 3 Center for Primary Health Care Research, Region Skåne, Malmö, Sweden

**Keywords:** Health inequities, Social determinants of health, Intersectionality, Sweden

## Abstract

**Background:**

Intersectionality theory combined with an analysis of individual heterogeneity and discriminatory accuracy (AIHDA) can facilitate our understanding of health disparities. This enables the application of proportionate universalism for resource allocation in public health. Analyzing self-rated health (SRH) in Sweden, we show how an intersectional perspective allows for a detailed mapping of health inequalities while avoiding simplification and stigmatization based on indiscriminate interpretations of differences between group averages.

**Methods:**

We analyzed participants (*n*=133,244) in 14 consecutive National Public Health Surveys conducted in Sweden in 2004–2016 and 2018. Applying AIHDA, we investigated the risk of bad SRH across 12 intersectional strata defined by gender, income and migration status, adjusted by age and survey year. We calculated odds ratios (with 95% confidence intervals) to evaluate between-strata differences, using native-born men with high income as the comparison reference. We calculated the area under the receiver operating characteristic curve (AU-ROC) to evaluate the discriminatory accuracy of the intersectional strata for identifying individuals according to their SRH status.

**Results:**

The analysis of intersectional strata showed clear average differences in the risk of bad SRH. For instance, the risk was seven times higher for immigrated women with low income (OR 7.00 [95% CI 6.14–7.97]) than for native men with high income. However, the discriminatory accuracy of the intersectional strata was small (AU-ROC=0.67).

**Conclusions:**

The intersectional AIHDA approach provides more precise information on the existence (or the absence) of health inequalities, and can guide public health interventions according to the principle of proportionate universalism. The low discriminatory accuracy of the intersectional strata found in this study warrants universal interventions rather than interventions exclusively focused on strata with a higher average risk of bad SRH.

## Background

Health disparities have now been documented by a wealth of epidemiological research for some decades [1, 2], but remain a pressing concern. Such inequalities have often been captured in the form of socioeconomic gradients [3, 4] whereby people in higher positions on the social ladder enjoy better health and quality of healthcare than people in lower positions. While socioeconomic position has received a large share of this research interest [5], disparities between groups defined by, i.e., gender [6], race, ethnicity, immigration status or racialization [7], have also been amply documented. However, some limitations to this body of research remain.

Studies of health inequalities have typically focused on one dimension at a time, such as socioeconomic position or gender, thus paying inadequate attention to how such dimensions may intersect. Meanwhile, a large share of the health disparities research has typically construed inequalities in terms of different levels of risk located in or borne by individuals or groups, rather than addressing dynamics *between* individual or groups [8, 9] or processes through which inequalities are produced [10]. Furthermore, health disparities research has been critiqued for insufficiently considering heterogeneity, through focusing almost exclusively on group average risk rather than on variations within and overlaps between groups. This may contribute towards simplification or essentialization of differences between groups, as well as to unjust stigmatization of “high-risk” groups or individuals [11, 12].

Due to its potential to address these concerns, intersectionality theory has increasingly been promoted and adopted in quantitative health disparities research e.g., [10, 13–15]. In this study, we apply an intersectional perspective combined with an analysis of individual heterogeneity and discriminatory accuracy (AIHDA) [11, 16, 17] to the investigation of disparities in SRH in Sweden. This is done in order to obtain a more detailed mapping of health inequalities while mitigating simplification and stigmatization based on indiscriminate interpretations of differences between group average risk.

### An intersectional perspective on population health research

Intersectionality theory, articulated and advanced by theorists including Crenshaw [18] and Hill Collins [19], centers on the understanding of social categorizations such as gender, class and race/ethnicity/racialization as being interconnected rather than separate, and as creating overlapping and interacting systems of discrimination or disadvantage. The principal idea is thus that the social categorizations conditioning the distribution of resources and power, and thus health, need to be considered as interlinked rather than as unidimensional. In the context of quantitative population health research, an intersectional perspective thereby motivates the study of strata defined by the combination of several socioeconomic dimensions (e.g., age, gender, income, racialized identity and sexual orientation), contrasting with conventional analysis of socioeconomic gradients in health often based on singular dimensions. In this manner an intersectional perspective can improve the mapping of inequalities in health and therefore better illustrate patterns of disadvantage.

Such improved mapping of disparities fits well within the current movement towards precision public health [20]. Relatedly, it can support the implementation of proportionate universalism, as formulated by Marmot and Bell [4], in public health resource allocation. According to this principle, interventions aiming to ameliorate health disparities should be directed at the whole population (i.e., be universal) but be combined with targeted actions of a scale and intensity proportional to the level of disadvantage in specific population groups. For decision-making about whether or how universal interventions should thus be accompanied by targeted ones, improved knowledge about existing health disparities is of central importance.

Furthermore, applying an intersectional perspective means directing interest towards the dynamics of power and wealth distribution in society, rather than to levels of risk as attributes of individuals or groups, in the interest of facilitating the amelioration of health inequalities through social change [21, 22]. Accordingly, the intersectional strata constructed in this study should be considered in terms of social contexts [23] rather than as characteristics of individuals. This can mitigate the risk of excessive biomedical reductionism threatening current precision-based public health [24], while reducing the likelihood of “blaming the victim” as frequently discussed when investigating socioeconomic differences conceptualized at the individual level.

In an influential classification of intersectional research, McCall [25] distinguishes between anti-, inter- and intra-categorical approaches (for further discussion see [26]). Epidemiology principally consists of the quantitative analysis of average differences between demographic, socioeconomic and biomedical population groups, and thereby per se adopts an inter-categorical (henceforth referred to as categorical) approach. However, the population categories under study should also be evaluated in relation to their discriminatory performance as classifiers, i.e., their capacity to accurately classify the individuals according to the health outcome of interest [26, 27]. Such evaluation, complementing the information on average risk differences, can serve to prevent the “tyranny of the averages” [12] through which the same average value is attributed to all the members of a group without considering the individual heterogeneity around the group average or any overlap between categories. If the discriminatory accuracy (DA) is low, the validity or relevance of the categorization for risk assessment or targeted intervention can be questioned in relation to the outcome at hand. In this sense, an anti-categorical stance can be adopted. This is important for the purposes of avoiding simplification or essentialization of differences between groups, alongside under- or overtreatment and ineffective public health interventions [17].

### Self-rated health

Measures of self-rated or self-assessed health, through which individuals are asked to evaluate their own health status, typically on a four- or five-point scale, are widely used in population health research. In terms of what it actually assesses and how it is linked to objective medical outcomes, this measure is not entirely understood [28]. It is subjective, non-specific, and encompasses cognitive, cultural and medical, or social and biological, dimensions [29]. However, its associations with mortality have been repeatedly demonstrated for different population groups and in various countries including Sweden [29–31]. In fact, its predictive power has been noted to often be stronger than that of more objective medical factors [32].

Inequities in SRH have been documented both internationally [33] and in Sweden [34–38], between socioeconomic groups [36, 37], genders [38] and individuals with or without an immigrant background [34, 35]. Some studies on SRH disparities have used intersectional approaches. For example, the impacts of class, gender, race, migration and sexual orientation in Canada have been investigated [39, 40], as have those of gender, sexual orientation and race in the United States [41, 42], and of class, gender and regional context in Spain [43]. Common to these studies, however, is that they adopt a categorical approach focused on between-group differences in average risk, without assessing individual heterogeneity and thus potentially allowing for a complementary anti-categorical stance.

In the present study, we use intersectional categorization combined with an analysis of individual heterogeneity and DA (AIHDA), in order to improve our understanding of inequalities in SRH, related to income, gender and immigration status in Sweden. Our focus lies on income, gender and immigration status partially due to the possibilities and constraints of the National Public Health Surveys (NPHS) data, but mainly because these dimensions correspond with the categories perhaps most typically included in the intersectional study of health disparities: gender, class and race [44]. While immigration status only loosely correlates with concepts of race or ethnicity, conflating issues related to racialization, migration and citizenship [13], which also concern groups in Sweden other than first-generation immigrants, immigration status is a categorization central to processes of racialization in contemporary Sweden [45].

## Methods

### Study population

The study is based on data from 14 consecutive National Public Health Surveys (NPHS) performed in Sweden during 2004–2016 and 2018 [46, 47]. The NPHS are produced by the Public Health Agency of Sweden and the Swedish regions, and they gather self-reported information on health, lifestyle and living conditions. Between 2004 and 2016 the surveys were performed annually, each time comprising a random sample of 20,000 individuals aged 16–84 years. Since 2016 the survey has been conducted biannually, with a random sample of 40,000 individuals. Response rates range from 60.8% in 2004 to 42.1% in 2018 [48].

**Fig. 1:**
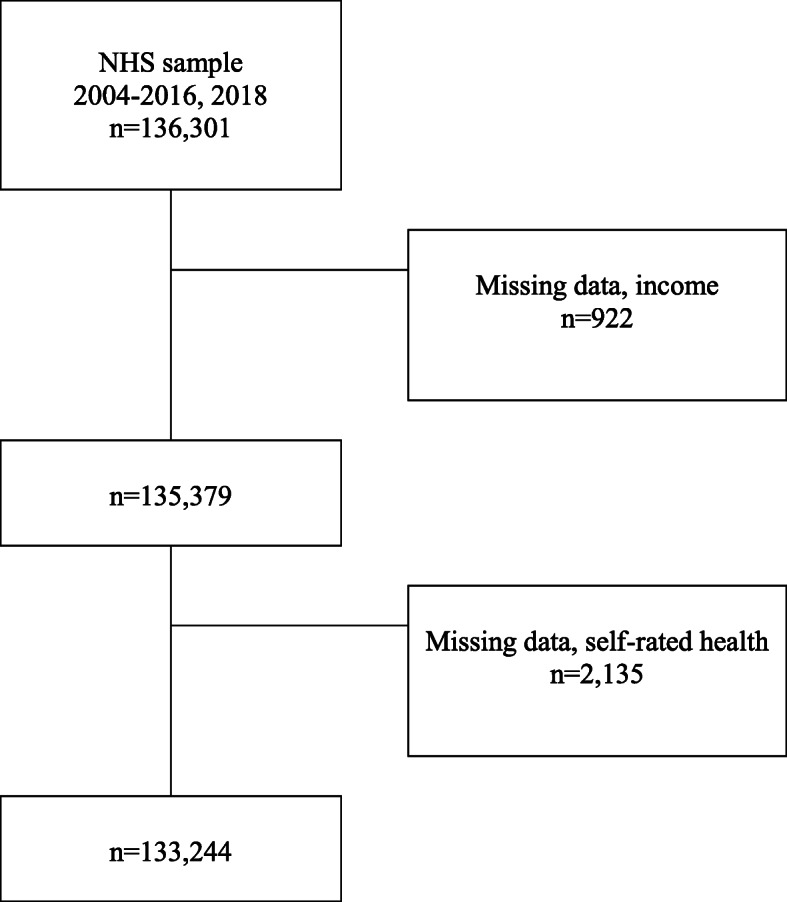
Study population flowchart. From the original population consisting of data from respondents to 14 National Health Surveys (2004–2016, 2018), we excluded those with missing data on income or self-rated health (SRH), obtaining a final sample of 133,244 people

Data from the 14 most recent surveys were pooled, resulting in a study population of 136,301 individuals. From this sample we excluded people with missing information on income (*n*=922) or self-reported health (*n*=2135). Thus, the final study population consisted of 133,244 individuals, i.e., 97.8% of the initial sample (Fig. 1).

The study was approved by the Swedish Ethical Review Authority (2019–01793) and the Data Safety Committee at the Public Health Agency of Sweden.

### Variables

Our outcome variable was *self-rated general health*, as assessed by the NPHS participants, based on responses to the question “How do you assess your general state of health?”, according to a five-point scale (very good; good; fair; bad; vary bad). The response options were recoded into good (very good; good) and bad (fair; bad; very bad), in accordance with the most commonly used coding of the variable [47]. The average prevalence of bad self-rated general health has been relatively stable over the survey years, although some fluctuations are present (see Table 1). Due to these fluctuations, our analyses adjust for survey year.

**Table 1:** Prevalence of bad self-rated health (SRH) among participants in the National Public Health Survey in Sweden, 2004–2016 and 2018

Year	Total	Bad SRH	
	N	N	%
2004	11,800	844	7.2
2005	5910	410	6.9
2006	5884	385	6.5
2007	5640	411	7.3
2008	10,935	687	6.3
2009	10,129	644	6.4
2010	9807	596	6.1
2011	9541	560	5.9
2012	9647	514	5.3
2013	9481	555	5.9
2014	9335	509	5.5
2015	9539	473	5.0
2016	9242	475	5.1
2018	16,354	1012	6.2

*Gender* was classified as a binary variable distinguishing between men and women, as these were the only response options provided by the NPHS questionnaire.

The information on household *income* was provided by the NPHS and divided the income data into tertiles (low; medium; high income). The high-income group encompassed the 20% highest reported incomes, the low-income group comprised the 20% lowest reported incomes and the medium-income group included the remaining 60% of reported incomes.

We classified *immigration status* as native, i.e., born in Sweden, or immigrant, i.e., born in another country.

As a way of operationalizing intersectional contexts, we created 12 *intersectional strata* by combining the two categories of gender, the three income categories and the two categories of immigration status. We used native men with a high income as the reference in the comparisons, as this group was assumed to occupy the position of greatest structural privilege.

In the analysis we adjusted for age, categorized as groups 16–24; 25–34; 35–44; 45–54; 55–64; 65–74; >=75 years), and for survey year entered as a categorical variable using 2004 as reference.

### Statistical analyses

Following a methodological approach described elsewhere [15, 16] we performed an intersectional AIHDA, in this case consisting of five consecutive logistic regressions modelling bad SRH as the dependent variable. We obtained odds ratios (OR) and their 95% confidence intervals (CI). In order to certify the validity of the AIDHA model used in the current dataset, we performed the estimations using bootstrap with 1000 repetitions [49].

The first regression (Model 1) included only age and survey year. The following models successively added gender (Model 2), income (Model 3), and immigration status (Model 4). Finally, Model 5 included the same variables as Model 4 but in the form of intersectional strata.

To qualify the socioeconomic and intersectional inequalities we complemented information on differences between average risks (i.e., the ORs) with measurements of their DA [16, 17]. For each model, we quantified the DA by means of the area under the receiver operator characteristics curve (AU-ROC) [27]. The AU-ROC measures the accuracy of the information provided by the variables in the model for discriminating individuals with bad SRH from those with good SRH. The AU-ROC takes a value between 0.5 and 1, where 1 indicates perfect discrimination and 0.5 means that the studied variables have no DA at all. Based on the classification provided by Hosmer and Lemeshow [50], we qualify the DA as: (i) “absent or very small” (AU-ROC=0.5–0.6), (ii) “small” (AU-ROC=0.6–0.7), (iii) “large” (AU-ROC=0.7–0.8) and (iv) “very large” (AU-ROC>0.8).

We further calculated the incremental change in the AU-ROC value (Δ-AUC), using Model 1 as reference. The Δ-AUC quantified the improvement in the DA obtained by a model, in relation to a reference model. The intersectional strata used in Model 5 allowed us to capture any statistical interaction of effects. If any such interaction existed, the DA of Model 5 would increase in comparison to that of Model 4.

We used IBM SPSS (Statistical Package for the Social Sciences) version 22 for PC and Stata 15.1 to perform all statistical analyses.

## Results

### Measures of average risk

As can be seen in Table 1, the prevalence of bad SRH in Sweden decreased slightly between 2004 and 2016 to then show a small increase in 2018. Overall, the median prevalence was 6.1% with a minimum value of 5.0% in 2015 and a maximum value of 7.3% in 2007.

Table 2 shows that the prevalence of bad SRH increased with age and with decreasing income. It was higher among women than among men and among immigrants compared to those born in Sweden. These latter findings can also be observed in the logistic regression analyses in Models 2, 3 and 4 (Table 3). Model 5 (see Table 3) displays a clear intersectional gradient. Compared with the reference stratum consisting of high-income native men, the risk of bad SRH successively increased to reach a level 7.0 times higher in the strata comprising low-income immigrant women. This maximum level was preceded by a 5.6 times higher risk among low-income immigrant men, a 4.7 times higher risk among middle-income immigrant women and a 3.8 times higher risk among low-income native women.

**Table 2:** Prevalence of bad self-rated health (SRH) by age, gender, income, and immigration status in the study population of participants in the National Public Health Surveys in Sweden, 2004–2016 and 2018

	Participants	Bad SRH	
	N	N	%
**Age**			
16–24	12,450	405	3.3
25–34	16,000	566	3.5
35–44	19,813	945	4.8
45–54	22,207	1461	6.6
55–64	25,212	1903	7.6
65–74	24,159	1473	6.1
>=75	13,403	1322	9.9
**Gender**			
Women	72,641	4738	6.5
Men	60,603	3337	5.5
**Income**			
Low	44,223	4076	9.2
Medium	44,467	2578	5.8
High	47,554	1421	3.2
**Immigration status**			
Native	117,345	6398	5.5
Immigrant	15,899	1677	10.6

**Table 3:** Odds ratios (OR) and area under the receiver operating characteristics curve (AU-ROC) with 95% confidence intervals (CIs) obtained from five consecutive logistic regression analyses modelling self-rated general health as a function of gender, income and immigration status as well as their combination as intersectional strata. All models are adjusted for age and survey year

	Model 1*	Model 2	Model 3	Model 4	Model 5
		OR (95% CI)	OR (95% CI)	OR (95% CI)	OR (95% CI)
*Gender*					
Women		1.22 (1.16–1.28)	1.16 (1.10–1.21)	1.16 (1.11–1.21)	
Men		Reference	Reference	Reference	
*Income*					
Low			3.27 (3.05–3.50)	3.07 (2.87–3.27)	
Middle			1.92 (1.80–2.05)	1.86 (1.74–1.99)	
High			Reference	Reference	
*Immigration status*					
Native				Reference	
Immigrant				1.86 (1.76–1.96)	
*Intersectional strata*					
Men/High income/Native					Reference
Women/High income/Native					1.31 (1.16–1.47)
Men/High income/Immigrant					1.86 (1.46–2.37)
Men/Middle income/Native					1.97 (1.75–2.21)
Women/Middle income/Native					2.37 (1.14–2.66)
Men/Middle income/Immigrant					3.87 (2.28–4.56)
Women/High income/Immigrant					3.07 (2.53–3.73)
Men/Low income/Native					3.70 (3.30–4.15)
Women/Low income/Native					3.76 (3.37–4.19)
Women/Middle income/Immigrant					4.70 (4.04–5.46)
Men/Low income/Immigrant					5.60 (4.79–6.54)
Women/Low income/Immigrant					7.00 (6.14–7.97)
AU-ROC	0.60 (0.60–0.61)	0.61 (0.60–0.61)	0.66 (0.66–0.67)	0.67 (0.67–0.68)	0.67 (0.67–0.68)
Δ-AU-ROC	Reference	0.01	0.06	0.07	0.07

* Model 1 is adjusted for age and survey year. Δ-AUC: increment in the AU-ROC compared to Model 1

In comparison with the reference category, the intersectional strata comprising males showed a lower risk of bad SRH than those including females, in all income and migration status categories (e.g., immigrant men with a high income had a lower risk than immigrant women with a high income, etc.). Strata including natives had lower average risk than those encompassing immigrant, in all income and gender categories (i.e., native females with medium income had a lower risk than immigrant females with medium income, etc.). Similarly, strata comprising persons with a high income showed a lower risk than those including persons with a medium or low income, in all gender and immigrant status categories (i.e., low-income, native men had a higher risk than medium- and high-income native men, etc.).

The strata defined by high income presented the lowest risks. An exception was the strata comprising high-income immigrant women, who showed a risk 3.1 times higher than the reference stratum.

### Measures of discriminatory accuracy

As seen in Table 3, Model 1 indicates that the information on participant age and survey year had a “very small” DA (AU-ROC=0.60), according to Hosmer and Lemeshow’s [50] classification. The DA slightly increased across the successive models with 0.07 units but remained “small” (AU-ROC=0.67) when simultaneously adding all the socioeconomic and demographic dimensions as separate variables in Model 4. Including all the information in the form of intersectional strata in Model 5 did not improve the DA. This indicates an absence of (multiplicative) interaction of effects.

## Discussion

Contributing to an intersectional and thus more precise epidemiological perspective based on AIHDA [15, 26], our study provides an improved understanding of the socioeconomic and demographic distribution of bad self-rated general health in the Swedish population.

Investigating differences in average risk between socioeconomic and demographic groups, our study replicates previous findings using conventional methodological approaches and showing the existence of inequalities in bad SRH between categories of income, immigration status and gender. However, by adopting a stratified intersectional approach we provide a more nuanced map of inequalities that is normally hidden in conventional studies of socioeconomic differences in health. Thus, compared with the reference stratum comprising high-income native men, the risk of bad SRH successively increased across intersectional strata to a level 7.0 times higher in the strata encompassing low-income immigrant women. This maximum level was preceded by a risk 5.6 times higher among low-income immigrant men, a risk 4.7 higher among middle-income immigrant women, and a risk 3.8 times higher among low-income native women.

The results from the intersectional AIHDA show the salience of gender, immigration status and income as stratification forces in Sweden [13]. At the same time, the analysis reveals that the impacts of these dimensions on the outcome are affected by each other [13]. While all strata comprising women had a higher average risk than those including males with the same income and immigration status, strata comprising women showed both relatively low and very high risk of SRH. This is in correspondence with the foundational insight of intersectionality scholarship that the positions of gendered subjects are fundamentally mediated by factors including racialization and class [19]. Along similar lines, all strata encompassing immigrants had a higher risk than those including natives with the same income and gender. Meanwhile, whereas most strata comprising persons with immigrant status were among those experiencing very high risk, two strata – those including males and females with high income – carried a lower risk. This shows that the relevance of immigration status for SRH in Sweden is clearly affected by factors including gender and income. The association between higher risk and lower income was, however, quite consistent.

In addition, the intersectional AIHDA goes beyond conventional analysis based on probabilistic measurements of average risk (e.g., odds ratios, prevalence ratios or relative risks) [11]. AIHDA stresses the relevance of incorporating complementary information on DA when evaluating differences between groups’ average risks, thereby taking into account the individual heterogeneities within, and overlap between, intersectional strata. In our study, the accuracy of the socioeconomic and demographic information for discriminating individuals with bad SRH from those with good SRH was low. This small DA expresses the existence of false negatives (i.e., individuals with bad SRH in strata with a low average risk for bad SRH) and false positives (i.e., individuals with good SRH in strata with a high average risk for bad SRH). Our study thereby provides a detailed mapping of health disparities alongside complementary information on its DA, and yields improved information for potential public health interventions. From an ethical point of view, focusing on specific population groups because of their higher risk of ill-health may convey a risk of stigmatization, but, on the other hand, the benefits of focused public health interventions may outweigh that harm. Using an AIHDA approach, we can better evaluate to what degree a universal intervention needs be proportionately targeted to specific groups with high average risk. A low DA supports universal intervention while a high DA merits targeted efforts. In this way, intersectional AIHDA can contribute to precision public health within Marmot’s framework of proportionate universalism [4, 51].

The Δ-AUC in Model 5 revealed no statistical interaction between the variables comprising the intersectional strata. This finding might appear counterintuitive as intersectionality theory refers to “the *interaction* between gender, race, and other categories” [52], p 68. It is worth stressing here that the concepts of intersectional strata and interaction are used somewhat differently in qualitative intersectionality research than in quantitative (social) epidemiology. Typically, intersectionality research considers the dimensions making up an intersectional identity as being not only interconnected but inseparable. In addition, the interaction of effects (not necessarily equaling the sum of the individual dimensions) pertaining to a certain outcome is not necessarily quantified. In quantitative (social) epidemiology, the effects of the different dimensions that define the intersectional strata can be disentangled, as in our Models 2–4 [26]. Furthermore, the health effects of inhabiting a particular intersectional stratum can be decomposed into additive and interaction effects. Hence, the concept of quantifiable (statistical) interaction is thus much more specific. We argue that intersectional risk heterogeneities are relevant regardless of whether the underlying mechanisms are additive or interactive [26].

Our study could have been performed using *multilevel* AIHDA, i.e., MAIHDA, as described [11, 53] and implemented [17, 54, 55] elsewhere. While both AIHDA and MAIHDA conceptually consider the intersectional strata as contexts, the MAIHDA has the advantage of providing a statistical technique that fits better with this concept. In addition, MAIHDA provides precision-weighted estimates of risk in strata with few individuals, as the strata estimations are shrunken towards the grand mean. Besides this, the multilevel approach uses the grand mean rather than a specific stratum as the reference in the intersectional comparisons [53]. A practical limitation of the MAIHDA is that being a multilevel random effects approach it is more suitable for implementation in large databases and particularly when analyzing many strata. In this study, we chose the AIHDA approach because the database was relatively small. A strength of the present AIHDA study is that it provides a valid and accessible alternative for quantitative intersectional analyses suitable for the monitoring of health inequities in routine public health surveys.

### Limitations

A limitation of this study lies in its observational and cross-sectional design, which allows for the study of correlations but does not enable the drawing of conclusions concerning causal relationships. Furthermore, the variables used infer certain limitations.

The outcome variable could have been recoded differently. The response option “fair SRH” was here included in the category of bad SRH, but, as noted by the Public Health Agency [47], an alternative strategy would be to instead consider it as a neutral category and not include it in bad or good SRH. It is furthermore possible that the interpretation of the concept of “fair SRH” can differ between groups. While this represents a limitation, our categorization of the SRH variable is the one most commonly used [47].

The outcome measure is self-assessed, and it is possible that different groups of people can have different reporting behavior [28], i.e., assess their health differently even if objective health measures are the same, and vice versa. The act of assessing one’s own health involves a cognitive process influenced by contextual factors including an individual’s understanding of the meaning and content of the concept of health, and of the frameworks for its evaluation [29]. Such understandings, frameworks and norms can obviously be historically and culturally contingent, and differences in reporting behavior between population groups have indeed been documented [29]. For example, elderly people in countries including Sweden [56] have been found to downplay functional limitations and chronic conditions when evaluating their health. This implies that measures of SRH are likely to overestimate the health of older persons [28, 57]. For this reason, we adjusted for age in our analysis instead of including age as an explanatory variable.

Differences in reporting behavior have been found between countries or cultures [29] and between groups with different socioeconomic status. Persons in lower socioeconomic groups have tended to rate their health more optimistically than persons in higher socioeconomic groups, as compared to “objective” health factors or diseases [28, 58, 59]. Women have also been found to be more optimistic than men about their health [28]. Such studies suggest that measures of SRH may overestimate the health of women and persons in lower socioeconomic groups, and thus underestimate health disparities. However, studies from Sweden found no significant differences in reporting of SRH between socioeconomic groups [31, 56]. While the latter studies support the comparability of the groups included in this study, the former studies suggest that any existing reporting bias is likely to under- rather than overestimate health inequalities.

As regards further limitations, our binary definition of gender was constrained by the response options offered by the NPHS, which disregards the more accurate continuum representation of both sex and gender [60]. Relatedly, from an intersectional viewpoint it would have been interesting to include dimensions such as sexual orientation, cis- versus transgender and functional diversity, in order to improve the capturing of existing disparities and heterogeneities in SRH. However, data availability and the size of the database prevented the addition of such information. The inclusion of other dimensions, such as educational attainment and marital status, might furthermore have increased the DA of intersectional strata. Conversely, if the DA was high, this would remain unnoted in a conventional non-AIHDA analysis using any number of variables.

Furthermore, our categorization of immigrated versus native-born individuals can be seen as simplistic. It conflates issues related to racialization, citizenship, migration and trauma [13], disregarding differences between groups such as refugees and immigrants from other Nordic countries [34], and excluding other groups that are affected by processes of racialization in contemporary Sweden, such as Sami populations, Jewish Swedes and second-generation immigrants. Previous research has indeed critiqued the use of the category of immigrated versus native-born in the study of SRH in Sweden, as it encompasses large variety within and overlap between groups [61]. Still, immigration status is central to processes of racialization in contemporary Sweden [45]. While pointing to the “bad SRH” of certain population groups might be overly simplistic, essentializing and in alignment with xenophobic tendencies [62], the neglect of any existing health disparities can also prevent societal changes toward improvement. Considering the DA of the immigration status dimension and of the intersectional strata in relation to the studied outcome aims to help resolve this dilemma [12, 61].

The purpose of this study was not to generate a predictive model which can be applied across contexts, but rather to show the distribution of risk for bad SRH between intersectional strata in Sweden during the studied time period. Still, in order to avoid an overfitting of the AIDHA model as used in the current dataset, we performed a bootstrap analysis with 1000 repetitions [49].

As the study rests on the pooling of data from consecutive surveys, there is a theoretical possibility that some individuals may have participated in more than one survey. However, as a national random sample was created for each survey, the probability of any such multiple measurements having had a considerable effect on the results is very small.

Finally, while intersectionality theory originates in qualitatively and theoretically oriented research, and some researchers question its commensurability with quantitative analysis [63], we side with others who have pointed to the importance of applying intersectional approaches in quantitative population health research [13–15].

## Conclusion

By mapping interrelating socioeconomic contexts and assessing the presence of individual heterogeneity through measures of DA, intersectional AIHDA offers a fruitful analytical approach for the investigation of health inequalities, in accordance with the principle of proportionate universalism. Analyzing average risks, we found a clear social gradient of self-rated general health. That is to say, our results point towards the effects of the unequal distribution of resources and power between groups defined by gender, immigration status and income on SRH in Sweden. At the same time, the low DA indicates substantial individual heterogeneity within strata. Our findings speak against focusing interventions only on specific strata as this would be ineffective and could potentially lead to unnecessary stigmatization. Interventions towards improving SRH in Sweden should thus be universal and encompass addressing the unequal distribution of resources which gives rise to health disparities.

## Data Availability

The dataset analyzed during the current study is available from the Public Health Agency of Sweden [46] on request.

## Abbreviations

AIHDA: Analysis of Individual Heterogeneity and Discriminatory Accuracy
AU-ROC: Area Under the Receiver characteristic Curve
CI: Confidence Interval
DA: Discriminatory Accuracy
MAIHDA: Multilevel Analysis of Individual Heterogeneity and Discriminatory Accuracy
NPHS: National Public Health Survey
PR: Prevalence Ratio
SRH: Self-Rated Health
Δ-AUC: Incremental change in the AU-ROC value
